# Numerical Investigation on the Structural Behavior of a Short-Span Cable-Stayed Bridge with Steel and CFRP Hybrid Cables

**DOI:** 10.3390/ma17092032

**Published:** 2024-04-26

**Authors:** Chunling Lu, Xiangxiang Wang, Yuwen Ning, Kang Wen, Qiang Wang

**Affiliations:** 1 Guangxi Key Laboratory of Green Building Materials and Construction Industrialization, Guilin University of Technology, Guilin 541004, China; 2000013@glut.edu.cn; 2 College of Civil Engineering, Guilin University of Technology, Guilin 541004, China; 2120210769@glut.edu.cn (X.W.); 1020220681@glut.edu.cn (Y.N.); 2120200703@glut.edu.cn (K.W.); 3 Collaborative Innovation Center for Exploration of Hidden Nonferrous Metal Deposits and Development of New Materials in Guangxi, Guilin University of Technology, Guilin 541004, China

**Keywords:** short-span cable-stayed bridge, steel and CFRP hybrid cables, CFRP cables, static and dynamic behavior, finite element analysis

## Abstract

In this paper, a thorough investigation is presented on the static and dynamic behaviors of a short-span cable-stayed bridge (CSB) incorporating steel and carbon fiber reinforced polymer (CFRP) hybrid cables. The study focuses on the world’s largest span and China’s first highway, CFRP CSB. The performance of the CSB was compared using numerical simulations under four different cable patterns: steel cables, CFRP cables, and steel, and two types of hybrid cables with different structural arrangements. The results indicate that the use of the use of CFRP cables in the long cable region in the short-span CSB project investigated in this study offers improved performance in terms of stability, seismic response, and reduced displacements. In comparison to CFRP cables, hybrid cables have demonstrated a reduction of 12% in the maximum vertical displacement of the main girder. On the other hand, the hybrid cables result in reduced maximum internal forces and longitudinal and lateral displacements of the main girders and towers compared to steel cables. The difference in the arrangement of CFRP cables in the long cable region or short cable region is not obvious under dead loads, but significant differences still exist between the CFRP cable bridges in the short cable region and the long cable region in terms of live load effects, temperature effects, and dynamic characteristics.

## 1. Introduction

Currently, cable-stayed bridges (CSBs) extensively utilize steel cables. However, the significant self-weight and sag of these conventional cables make them more prone to causing decreased longitudinal stiffness and increased nonlinear effects on the bridge. Consequently, this reduces the carrying efficiency of cable-stayed bridges. Additionally, traditional steel cables exhibit poor corrosion and fatigue resistance [1]. The replacement of steel cables not only impacts traffic but also escalates maintenance costs in the long run. In comparison to steel cables, carbon fiber-reinforced polymer (CFRP) cables possess several advantages. They are lightweight, possess high strength, and exhibit corrosion resistance. Additionally, they offer excellent properties such as good fatigue resistance [2], low relaxation [3], and suitable creep behavior [4,5]. These properties enhance the service life of cables and reduce maintenance costs during the operation of CSBs. Liu et al. demonstrated through a comparison of the lifecycle costs between a steel CSB and a CFRP CSB that the CFRP CSB can achieve lower lifecycle costs than the steel CSB due to its lower repair costs and user costs [6]. In a pioneer work by Meier [7,8], the application of CFRP materials to CSBs was discussed, and the great idea and theoretical feasibility of constructing an 8400m-span CFRP cross-sea CSB were proposed and discussed. Out of the 24 cables in the world’s first CSB with CFRP cables in Winterthur, only 2 cables were composed of CFRP, while the remaining cables were constructed using high-tensile steel strands [9]. An experimental CSB with CFRP cables—Jiangsu University footbridge—was also built in China [10]. Xie et al. [11] and Zang et al. [12] studied the static and dynamic characteristics of short-span cable-stayed footbridges with CFRP cables. At present, CSBs with CFRP cables are mostly employed in the structure of footbridges, but lighter loads cannot give full play to the advantages of CFRP cables. It is important to consider the static and dynamic behaviors of short-span CSBs with CFRP cables under the influence of vehicle load, temperature load, and seismic oscillation.

Due to the excellent performances of CFRP cables, the study of CSBs with CFRP cables has gained significant attention. Ren [13] proposed a cable-stayed bridge using CFRP cables and UHPC components and quantitatively analyzed it based on factors such as cross-sectional stiffness, impact resistance, local stability, and shear resistance. Wang et al. [14] evaluated the safety factor and applicable length of FRP cables, demonstrating the potential for their application in long-span cable-stayed bridges. Adanur et al. [15] and Gunaydin et al. [16] analyzed the dynamic performance of the Jindo Bridge using CFRP stay cables subjected to seismic load through numerical simulations. Xie et al. [17,18] examined the static-dynamic characteristics of various cable-stayed bridges with CFRP cables. Mei et al. [19] discovered that under the action of temperature load, the CSBs with CFRP cables would generate large internal forces due to the inconsistency of CFRP cables with girder and pylon deformations. Furthermore, the lower elastic modulus and higher price of CFRP cables make it impossible to broadly utilize CFRP cables in long-span CSBs [2,20,21] like traditional cables. Additionally, the anchorage of CFRP cable presents a significant challenge [22,23]. Due to the orthotropic characteristics of CFRP materials, the stress concentration caused by traditional cone anchors clamping the anchorage region can lead to premature failure of CFRP tendons. Therefore, some scholars have improved the filling and structure of CFRP tendon anchorage systems to enhance the bearing capacity of CFRP cable anchorage [24,25,26,27,28].

In recent years, some scholars have started to examine the use of composite cables in CSBs as an alternative to address the shortcomings associated with CFRP cables. Current research on hybrid cables mainly focuses on hybrid cross-section stay cables and hybrid structure stay cables. Xiong et al. [29] and Cai et al. [30] highlighted the economic benefits and the improved static and dynamic behavior associated with the use of combined section cables consisting of CFRP and steel in CSBs. However, the manufacturing difficulties and the susceptibility of steel wires to corrosion still pose challenges for hybrid cross-section cables, as the steel wires in these cables can be corroded. Additionally, the design of anchorage systems for hybrid cross-section cables is far from being able to support practical engineering applications. Yang et al. [31] introduced a hybrid arrangement of FRP tension cables specifically designed for cable-stayed bridges with main spans exceeding 2000 m, offering a suitable solution for such large-span structures. Hybrid structural cable-stayed bridges achieve complementary advantages by arranging cables made of different materials in different regions of the structure. Currently, research on the mechanical properties of CFRP cables and anchorage systems is relatively more mature. Although there have been instances of CFRP cable applications in short-span cable-stayed bridges, there is still limited research on the performance of CFRP cables in short-span cable-stayed bridges.

This study aims to overcome the limitations of prior research by specifically examining the ongoing construction of China’s first highway CSB using CFRP cables. A comprehensive explanation of the hybrid cables’ design method is provided, considering the mechanical properties of both steel and CFRP cables. Finite element models are created and compared for CSBs with steel cables, CFRP cables, and two different cable arrangement forms. This study conducts an analysis of the mechanical properties of four cable patterns under various loads, including dead load, live load, and temperature load. Furthermore, an evaluation is carried out to assess their dynamic characteristics and seismic responses. The study aims to verify the feasibility and rationality of using steel and CFRP hybrid cables in short-span CSBs, considering their static and dynamic behaviors.

## 2. Introduction of Tuhaihe Bridge

**Figure 1:**
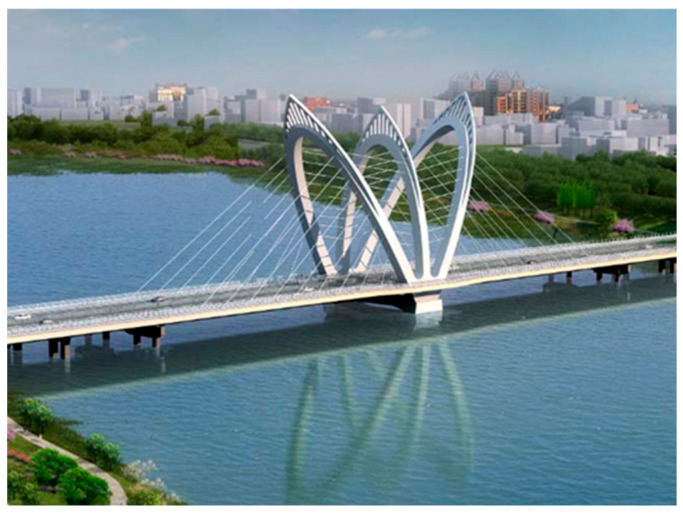
Design sketch of CSB.

**Figure 2:**
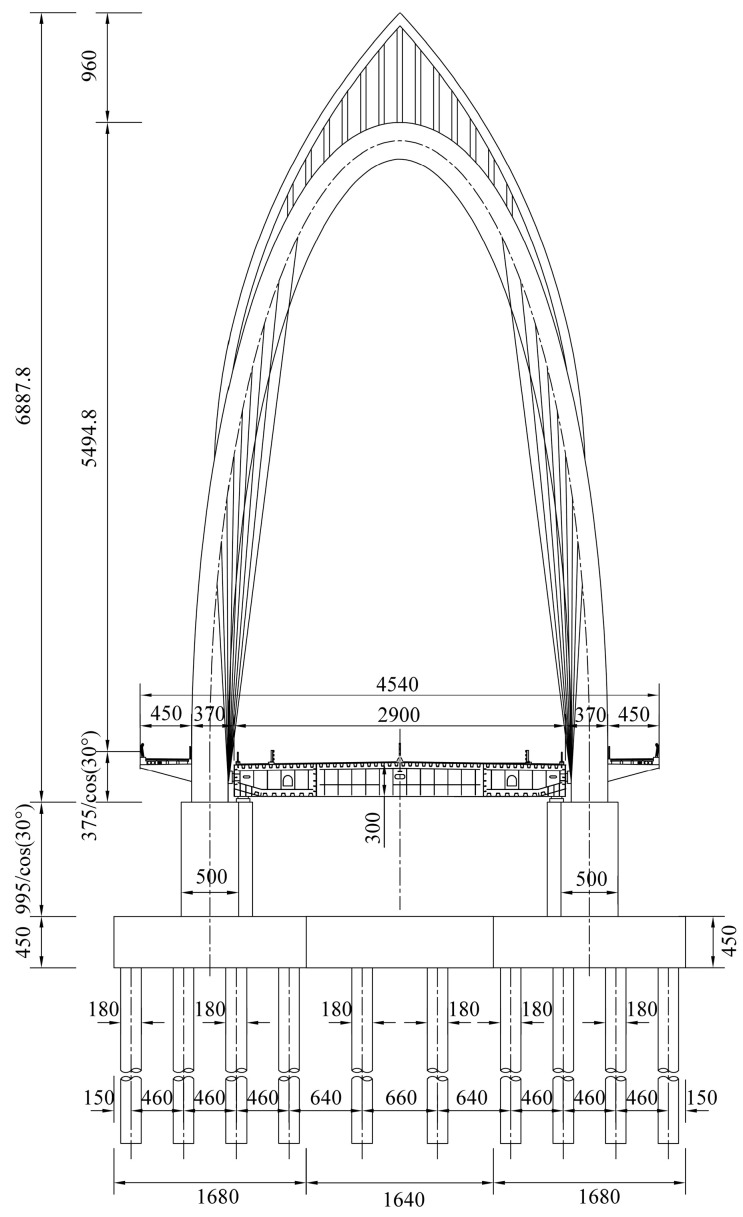
Main bridge cross-section (unit: cm).

**Figure 3:**
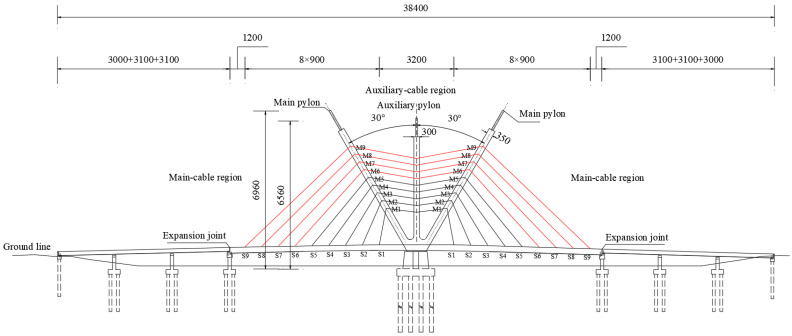
Main bridge general layout (unit: cm).

Tuhaihe Bridge, situated in Liaocheng City, Shandong Province, China, is the first highway CSB using CFRP cables in China and the largest highway CSB using CFRP cables worldwide. The bridge’s span arrangement consists of (30 + 31 + 31) m + (100 + 100) m + (31 + 31 + 30) m. The main bridge encompasses a 200 m-long two-span semi-floating CSB configuration featuring a single pylon and a dual cable plane designed in a lotus shape. Furthermore, the approach bridges consist of 92 m-long three-span cast-in-situ prestressed concrete continuous box girder bridges. The main girder of the main bridge is constructed from Q345 steel, featuring a fully welded steel box girder design with a single box and double chambers configuration. The box girder exhibits a top width of 40 m, a girder height of 3 m at the center line, and a cantilever plate length of 5.5 m. The bridge pylon is a special-shaped three-arch pylon composed of main and auxiliary pylons. The main pylon on both sides is 52.211 m high above the bridge deck, the middle auxiliary pylon is set vertically with a height of 47.65 m, and the angle between the axis of the main pylon column and the auxiliary pylon column is 30°. The pylon body itself is fabricated using Q345 steel, utilizing a variable cross-section steel box section. The bridge incorporates a total of 36 pairs of spatial double cable plane stay cables arranged in a fan-shaped manner along the bridge. The main bridge’s foundation consists of a bearing platform constructed with C35 concrete and cast-in-place pile foundations made of C50 concrete. The design sketch and layout of the CSB are presented in Figure 1, Figure 2 and Figure 3.

The technical standards of the bridge are as follows. ① Design reference period: 100 years. ② Road grade: two-way, 8-lane urban secondary trunk road. ③ Design speed: 50 km/h. ④ Vehicle load: M-A loading. ⑤ Standard axle load for pavement design: BZZ-100. ⑥ Crowd load: take values according to Article 7.0.8 of the Technical Standard of Highway Engineering JTGB01-2014 [32]. ⑦ Wind Load: According to the provisions of the “Code for Loadings on Building Structures” (GB 50009-2012 [33]), the basic wind speed in Liaocheng City is 26 m/s, and the corresponding design wind speed is 36 m/s. ⑧ Seismic fortification criteria: 7-degree basic intensity and classification of design earthquake are the second group, seismic fortification category is classified as class A, and the seismic protection measure level is 8-degree.

## 3. Finite Element Simulation Technique

For this study, the Midas Civil finite element software 2021 V2.1 was employed to construct the finite element model of the main bridge. This software was chosen due to its ability to seamlessly incorporate bridge design specifications and generate calculation results that align with actual engineering.

### 3.1. Finite Element Modeling

**Figure 4:**
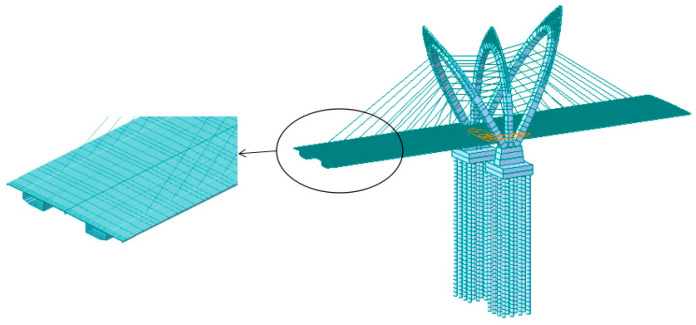
Finite element model of Tuhaihe bridge.

**Figure 5:**
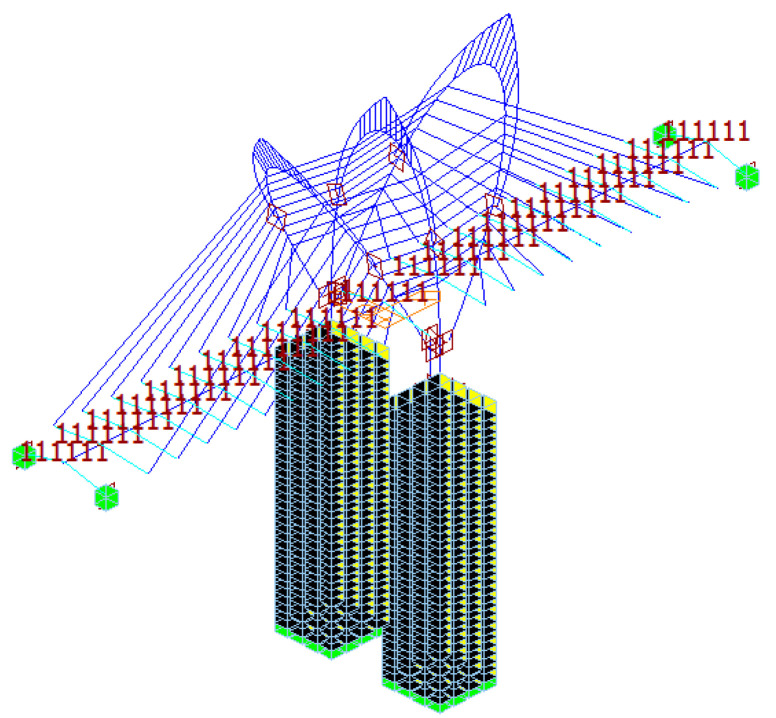
The boundary conditions of the CSB.

The main girder, bridge pylons, pylon base, abutments, and pile foundations of the CSB are simulated using beam elements. Truss elements are employed to model the stay cables. The simulation consists of a total of 1543 nodes and 1560 elements, as illustrated in Figure 4. In Section 3.3, the accuracy of the element division method is demonstrated using the completed Sutong Bridge as a case study. The model is constructed using the same method, dividing the cells, and then compared with the actual engineering test results. This comparison confirms the ability of the modeling method proposed in this paper to deliver precise calculation results. The boundary conditions are set as follows: the side-span support pivot of the main girder is supported in a general way, the stay cables are rigidly connected to the main girder, the main girder is elastically connected to the bridge pylon, the pylon supports and bridge pylons, and abutments and pile foundations are elastically connected. The pile foundation is elastically supported with the surrounding soil, and the pile bottom is supported in a general way. The boundary conditions in the completed bridge state are shown in Figure 5, and the specific boundary conditions are shown in Table 1. By incorporating the Ernst equivalent elastic modulus *E_eq_* [34], the impact of cable sag effects is taken into account while considering the influence of geometric non-linear effects such as beam-column effects and large displacement effects on the structure [35]. Detailed information on the main components of the main bridge model is shown in Table 2. The structural steel of the pylon and main girder uses Chinese standard Q345 steel [35]. The lower structures, including the pylon base, abutments, and pile foundations, predominantly consist of reinforced concrete. The material properties of these components adhere to Chinese standards [36]. Galvanized parallel steel wire cables [35] are used for the steel cables. The CFRP cables are constructed using standard CFRP tendons, with their material properties sourced from relevant literature [37].

The tensile strength of carbon fibers has significant randomness due to the presence of internal defects, and increasing fiber length increases the probability of material defects, thus significantly reducing its strength. Therefore, this paper utilizes the couple-Weibull model proposed by Huang [38] to estimate the reduced tensile strength of carbon fiber cables with different lengths, as shown in Equation (1).

(1) $$\tilde{\sigma }=\alpha \sigma_{1}L^{-\left\{\psi e^{\left[-\xi \left(L/L_{0}\right)\right]+C}\right\}/m_{1}}\Gamma (1+\frac{1}{m_{1}})+(1-\alpha )\sigma_{2}L^{-\left\{\psi e^{\left[-\xi \left(L/L_{0}\right)\right]+C}\right\}/m_{2}}\Gamma (1+\frac{1}{m_{2}})$$

where α represents the weight coefficient, $m_{1}$ and $m_{2}$ represents the shape parameter, $\sigma_{1}$ and $\sigma_{2}$ represents the characteristic stress, ψ and C represents the material test result parameter.

In this study, the cable length of the bridge is chosen to be within the range of 12.8 m to 122.8 m. According to Equation (1), it is calculated that for the 2400-grade CFRP cable with a length of 122.8 m, the stress attenuation caused by the length effect reduces the tensile strength of the material to 2000 MPa. However, since this study adopts the principle of equal cross-sectional area for the design of the composite cable-stayed bridge, its tensile strength is still higher than the tensile strength of the steel cable, which is 1770 MPa. Therefore, the influence of the length effect can be neglected when designing the replacement cable section.

### 3.2. Material Model

**Figure 6:**
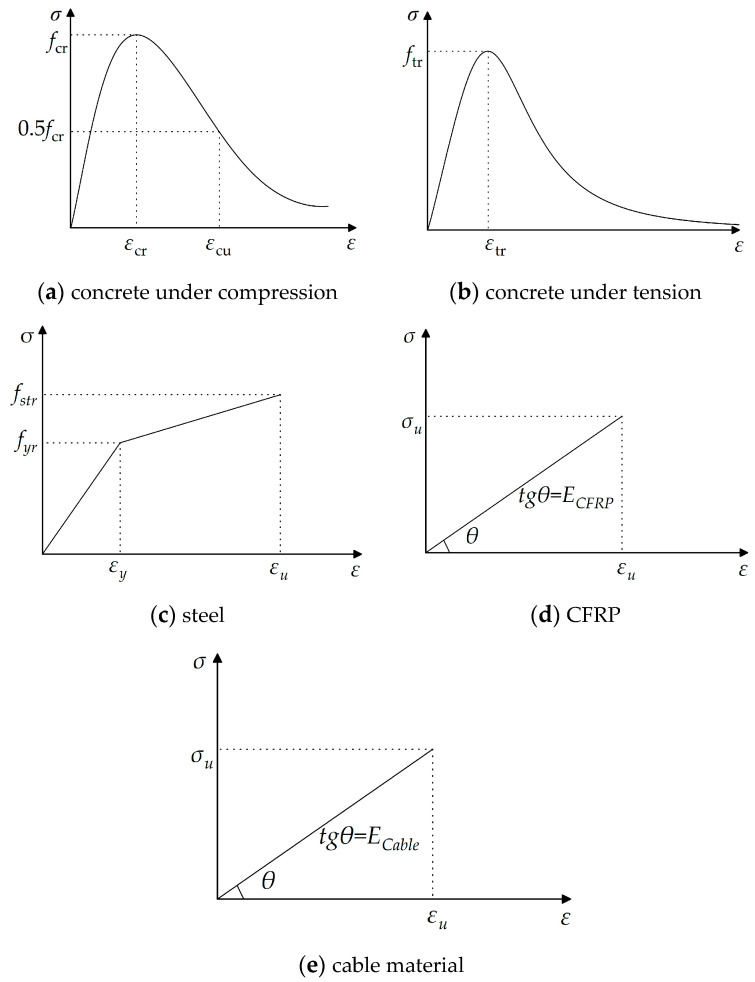
The stress-strain relationships of the materials in finite elements.

The stress-strain relationships of the materials used in the finite element analysis are illustrated in Figure 6. This relationship is derived from the specifications outlined as follows: the uniaxial stress-strain curves of concrete, in both compression and tension states, are determined based on the values specified in “Code for Design of Concrete Structures” GB50010-2010 [39]. The idealized stress-strain curve for steel is defined according to the “Design Standard for Steel Structures” GB50017-2017 [40]. CFRP reinforcement belongs to brittle material with apparent linear elastic characteristics. Thus, its constitutive model can be described by the stress-strain curve shown in Figure 6. Additionally, to ensure safety, the stress-strain relationship of cables is considered only during the elastic deformation stage and is represented by a proportional function. The failure of cable-stayed systems can be characterized as a brittle failure.

Where *σ_cr_* and *σ_tr_* represent the peak values of the strengths of concrete under uniaxial compression and tension, respectively; *ε_cr_* represents the peak compressive strain of concrete corresponding to *σ_cr_*; *ε_tr_* represents the peak tensile strain of the concrete corresponding to *σ_tr_*; *σ_yr_* is the representative value of the yield strength of the steel bar; *σ_str_* is the representative value of the ultimate strength of the steel bar; *ε_y_* is the yield strain of the steel corresponding to *σ_yr_*; *ε_u_* is the peak strain of the steel corresponding to *σ_str_*; *σ_u_* represents the ultimate tensile stress of cable material, and *ε_u_* represents the ultimate tensile strain of cable material. The specific parameter values can all be obtained from the aforementioned specifications.

### 3.3. Verification of Finite Element Modeling Methods

**Figure 7:**
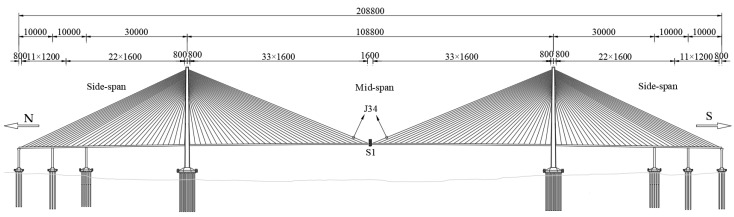
General layout (unit: cm).

This study centers around an under-construction short-span cable-stayed bridge (referred to as CSB), which lacks measured data for model validation. To overcome this limitation, the author chose the completed Su-Tong Yangtze River Highway Bridge (referred to as Sutong Bridge) as a case study to validate the accuracy of the finite element modeling method. Sutong Bridge’s main bridge structure is a semi-floating system consisting of a double-tower and double-cable-plane steel box CSB with auxiliary piers. The span arrangement is (100 + 100 + 300) m + 1088 m + (300 + 100 + 100) m. The main girder of the bridge is a closed, streamlined, flat steel box girder made of Q345 steel, featuring a top width of 34 m and a height of 4 m at the centerline. Transverse and longitudinal partitions are incorporated into the main girder, with top and bottom plates equipped with 6–10 mm thick U-shaped stiffeners. The bridge pylon follows an inverted Y shape, with a lower horizontal girder positioned below the main girder. The pylon stands at a height of 300.40 m, with a height above the bridge deck measuring 230.41 m. The height-to-span ratio is 0.212. Constructed using C50 concrete, the bridge pylon is a hollow box-shaped reinforced concrete structure. The diagonal cable adopts 1770 MPa parallel steel wire strands; the whole bridge has a total of 4 × 34 × 2 diagonal cables with eight specifications, whose cross-sectional area ranges from 5.35 to 12.05 × 10^−3^ m^2^, the maximum single cable length is about 577 m, the standard cable spacing is 16 m, and the cable spacing near the end of the side span is 12 m. The overall layout of Sutong Bridge is shown in Figure 7.

**Figure 8:**
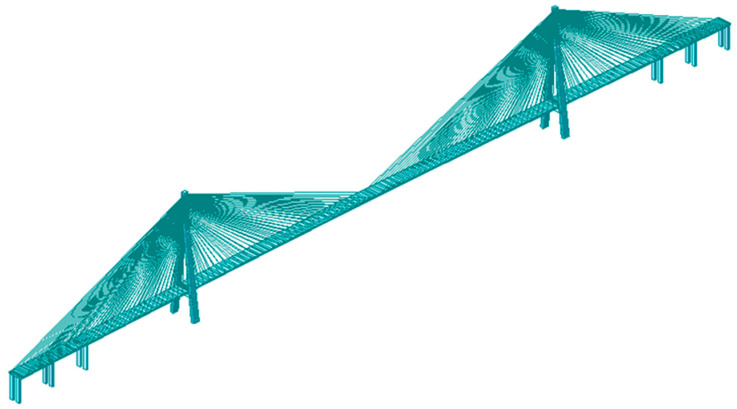
Finite element model of Sutong bridge.

The finite element model of the main bridge is shown in Figure 8, and the finite element modeling method is the same as in Section 3.1. The effect of cable sag was considered by incorporating the Ernst equivalent elastic modulus *E_eq_*. A rigid connection was assumed between the stay cables and the main girder. Five different mesh sizes are used for meshing the cable-stayed bridge, and a grid sensitivity analysis is conducted using NW-J32 as an example, as shown in Table 3. The calculation accuracy and efficiency are compared, and Mesh 3 is adopted as the baseline mesh for this study. The model comprised a total of 1035 nodes and 1018 elements. The boundary conditions in the bridge’s completed state are shown in Table 4.

**Figure 9:**
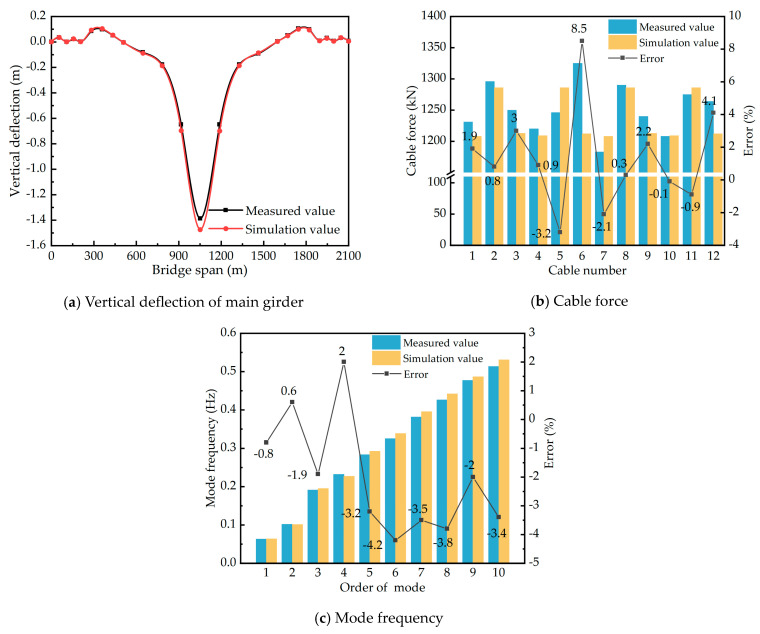
Comparison of measured and simulated values of Sutong Bridge.

Reference [41] conducted a comprehensive analysis of the mechanical behavior of Sutong Bridge in its completed state. To validate the accuracy of the Cable-Stayed Bridge (CSB) model, the numerical simulation results were compared with the measured data from Reference [41]. The study involved subjecting the CSB to a symmetrical load of 60 three-axle vehicles, each weighing 30 t. The maximum vertical deflection of the bridge deck was observed on the west side of the steel girder at the mid-span of the CSB (position S1), reaching a magnitude of 1388 mm. The deflection measurements were taken at various points, including the mid-span of the main girder, the 300 m span wind vent of the main girder, and the 100 m span bridge deck guardrail. A total of 26 measuring points were symmetrically arranged on both the east and west sides. Moreover, the cable forces of the CSB were evaluated using the ambient random vibration method [41]. The measured and simulated deflection results on the west side of the main girder and the cable forces of selected cables are presented in Figure 9a and Figure 9b, respectively. Additionally, Table 5 compares the dynamic characteristic analysis results of Sutong Bridge obtained in this study with those reported in Reference [41].

The comparison between the measured and simulated vertical deflections of the main girder, as shown in Figure 9a, reveals a ratio ranging from 0.88 to 0.95. The measured and simulated results of the incremental forces of the stay cables, presented in Figure 9b, exhibit a close agreement, with a deviation of less than 10%. These findings suggest that the finite element model accurately captures the overall static performance of the Cable-Stayed Bridge (CSB), as evidenced by the close resemblance between the simulated and measured deflections and cable forces under dead load conditions. Furthermore, Figure 9c demonstrates that the vibration mode frequencies of Sutong Bridge, as simulated in this study, closely align with the results reported in the literature [39], with a deviation of less than 5%. This indicates that the finite element model effectively captures the dynamic characteristics of the CSB. Based on these verifications, it can be concluded that the modeling method employed in this research is accurate and reliable. The finite element model developed provides an accurate representation of the overall static and dynamic behaviors of the CSB.

## 4. Cables Scheme Design

**Figure 10:**
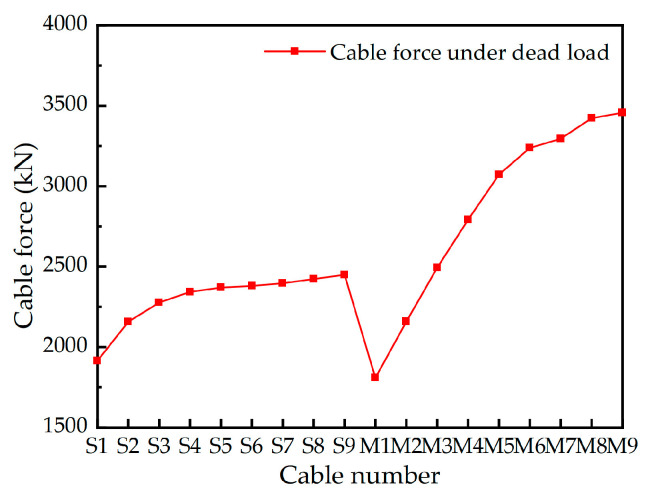
Cable forces under the design dead load.

The bridge as a whole is equipped with a total of 36 pairs of stay cables. Among these, the main cable sections comprise 2 × 9 pairs of stay cables connecting the main pylon to the main girder on each side, while the auxiliary cable sections consist of 2 × 9 pairs of stay cables linking the middle auxiliary pylon to the main pylon on each side. On the main pylon, the cable distance along the axial direction is 3.8 m, while on the auxiliary pylon, it is 3.0 m. On the girder, the cable distance is set at 9 m. The cable numbers and layout have been illustrated in Figure 3. Figure 10 gives the cable forces of stay cables under design dead load, where a total of 17,970.84 kN nodal load is applied as the self-weight of the diaphragm on the main girder and 150 kN/m secondary dead load.

### 4.1. Scheme Design of Steel Cables and CFRP Cables

Parallel wires with the tensile strength of 1770 MPa are employed for the cables of the CSB with steel cables (hereinafter referred to as steel cable bridge (SCB)), of which the steel cables at main-cable regions S1–S9 are all composed of 127φ7 galvanized steel wires, with a cross-sectional area of 4.89 × 10^−3^ m^2^, and the maximum cable length of a single cable in the main-cable regions is about 74 m. The steel cables at auxiliary-cable regions M1–M3, M4–M7, and M8–M9 in order are 121φ7, 151φ7, and 163φ7 galvanized steel wires, with a cross-sectional area of (4.89–6.28) × 10^−3^ m^2^, and the maximum length of a single cable in the auxiliary-cable regions is about 29 m. The material characteristics of steel cables refer to the Specifications for Design of Highway Reinforced Concrete and Prestressed Concrete Bridge and Culverts [35]. The values of these properties have been provided in Table 2.

In the context of replacing steel cables with carbon fiber-reinforced polymer (CFRP) cables, the principle of equal strength and equal stiffness is commonly applied. However, when using the equal strength principle, the higher tensile strength of CFRP cables leads to a smaller cross-sectional area compared to steel cables, resulting in a reduced overall stiffness of the bridge. On the other hand, the equal-stiffness principle may lead to large and uneconomical cross-sectional areas for CFRP cables. To address this, a compromise method is adopted in this study. The cross-sectional area of the CFRP cables is set to be the same as that of the steel cables, ensuring a balance between strength and stiffness. The safety factor for steel cables is taken as 2.5, while the safety factor for CFRP cables is approximately 3.4, as indicated by the parameters in Table 2 [41]. The CFRP cables of the CFRP cable bridge (hereinafter referred to as CFRPCB) use CFRP tendons with a standard tensile strength of 2400 MPa, in which the main and auxiliary cable regions are composed of CFRP tendons with a diameter of 7 mm and the number of carbon fiber reinforcement in each cable in the main and auxiliary cable regions is the same as the number of steel wires in the steel cables, and the material parameters of the CFRP cables are shown in Table 2.

### 4.2. Scheme Design of Steel Cables-CFRP Cables Hybrid

In order to comprehensively compare the static and dynamic responses of cable-stayed bridges when steel cables and CFRP cables are arranged in different positions, this study sets up two contrasting conditions: Condition 1 (HCB-1) with CFRP cables on the outer side and steel cables on the inner side of the CSB; and Condition 2 (HCB-2) with steel cables on the outer side and CFRP cables on the inner side. Section 5.2, Subfigure c shows the maximum deflection of the main girder of the CSB under live load for both the all-steel cable and all-CFRP cable schemes. It can be observed that under live load, there is a significant difference in the deflection of the main girder between the all-CFRP cable CSB and the all-steel cable CSB within the longitudinal range of 40 m–160 m. The deflection values are similar within a range of 40 m from both ends of the main girder. Therefore, this study selected a location 40 m away from both ends of the main girder to change the combination cable material. Different materials are used in the main cable zones S1–S5 and auxiliary cable zones M1–M5, as well as in the main cable zones S6–S9 and auxiliary cable zones M6–M9. Taking HCB-1 as an example, the cable arrangement diagram for the cable-stayed bridge with a combination of steel cables and CFRP cables is shown in Figure 3, with the CFRP cables marked in red.

## 5. Static Characteristic Analysis

### 5.1. Bridge Performances Due to Dead Load

**Figure 11:**
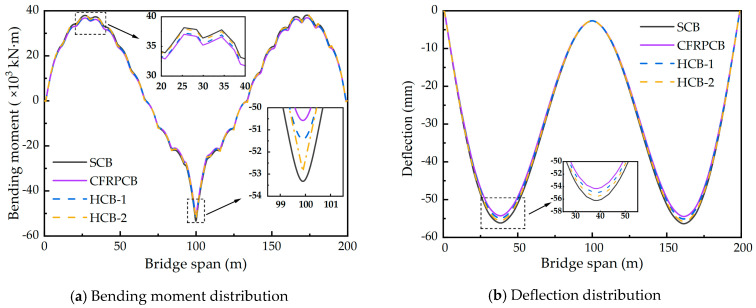
Distributions of bending moments and deflections of the main girder under the action of dead load. Note: The deflection is negative (positive) when the main girder bends downward (upward).

To assess the structural performances of CSBs with four cable patterns under dead load, the bending moment and deflection of the main girder should be analyzed and compared, as demonstrated in Figure 11.

In Figure 11a, the distribution trends of the bending moments in the main girder are nearly identical for all four cable patterns under dead load. The maximum negative bending moment occurs at the middle span of the main girder, while the maximum positive bending moment is observed over the side span. Comparing the three patterns, the SCB exhibits the largest positive and negative bending moments, measuring 38,158.3 and −53,739.9 kN·m, respectively. These values are 3.0% and 3.4% higher than those of the CFRPCB. The HCB falls between the SCB and CFRPCB in terms of bending moments. Figure 11b shows the deflection distribution trends for the four CSB patterns under dead load, with the maximum deflection occurring at both ends of the main girder. The maximum dead load deflections for the four patterns are −56.2 mm, −54.3 mm, −54.9, and −55.5 mm, respectively. The SCB exhibits the largest deflection at both ends of the main girder, which is 3.5% larger than that of the CFRPCB. The difference in bending moment and deflection of the main girder in the CSB for the four cable patterns is mainly caused by the weight difference between the steel cables and CFRP cables. Replacing the steel cables with lighter CFRP cables can reduce the self-weight of the upper structure of the CSB, which is beneficial for reducing the cost of the lower structure and improving the bridge’s load-carrying capacity. In the two cable combinations, replacing the steel cables with CFRP cables in the long cable region results in a greater reduction in weight, thereby achieving better deflection control.

### 5.2. Bridge Performances Due to Live Load

**Figure 12:**
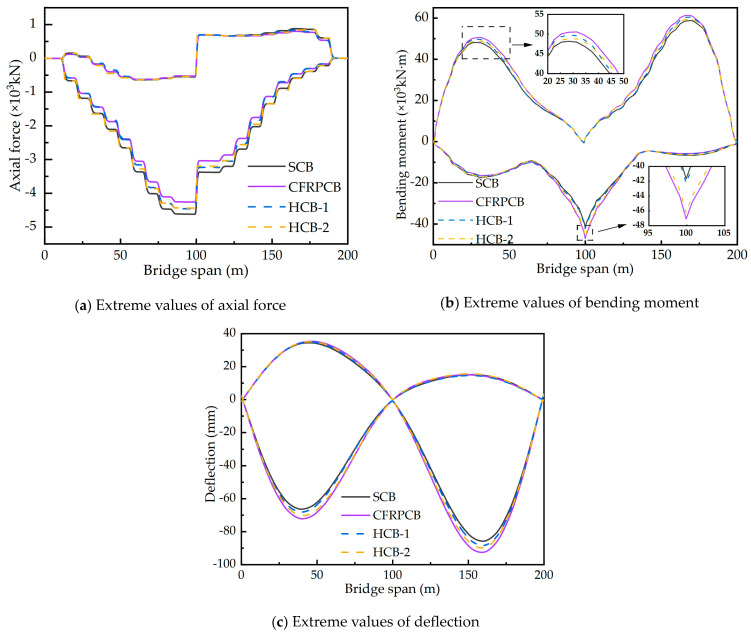
Extreme values of internal forces of the main girder of live load. Note: The axial forces of the main girder are positive in tension and negative in compression.

To investigate the structural performances of CSBs with four cable patterns subjected to live load, the extreme values of the axial forces, bending moments, and deflections of the main girder of CSBs with four cable patterns due to the load combination of vehicle load, crowd load, and wind load are analyzed and compared (see Figure 12). The wind load is decomposed into the transverse bridge wind load and the longitudinal bridge wind load, which are applied to the bridge structure.

Based on the analysis presented in Figure 12, the internal force distribution trends in the main girder of CSBs with four different cable patterns are found to be similar under live load. Due to the effect of wind loads along the bridge on the main and secondary pylons, there is a sudden change in axial force in the main girder at the midspan. Figure 12a specifically compares the extremes of axial forces in the four cable patterns under live load, with the maximum live load axial forces in the main girders measuring −4621.0 kN, −4261.7.8 kN, −4461 kN, and −4440.6 kN for the SCB, CFRPCB, and HCB, respectively. Compared to the SCB, the CFRP cable and the two hybrid cable patterns have reduced the maximum live load axial force on the main girder by 7.8%, 3.5%, and 3.9%, respectively. The axial force extremes in the main girder of the HCB fall between those of the SCB and the CFRPCB. This reduction in axial force can be attributed to the sag effect of the cables, which results in a decreased inclination angle at the girder’s end. Consequently, the horizontal component of the steel cables becomes larger than that of the CFRP cables, leading to an increase in the axial force of the main girder. The reduction of axial force in a CSB with a steel main girder can enhance the stability of the structure.

The results presented in Figure 12b,c demonstrate a close resemblance between the extreme values of bending moments and deflections in the main girder of the HCB and the SCB under live load conditions. Conversely, the main girder of the CFRPCB exhibits the largest extreme values of bending moment and deflection. This can be primarily attributed to the utilization of CFRP cables throughout the entire bridge, which leads to a reduction in overall bridge stiffness due to the lower elastic modulus of CFRP tendons. The maximum deflections under live load for the four cable patterns are recorded as −66.4 mm, −72.2 mm, 68.2 mm, and −70.2 mm, respectively. Compared to the CFRPCB, both the steel cable and hybrid cable patterns reduce the maximum deflections under live load by 7.6% and 5.0%, respectively. The maximum live load deflections of the HCB and the SCB are very similar. Therefore, the implementation of hybrid cables proves to be beneficial in improving the overall stiffness of CSBs compared to using CFRP cables exclusively. This allows for better structural performance and reduced deflection under live load conditions. Compared to the CFRPCB, the hybrid cable schemes reduce the maximum live load deflections of the main girder by 5.5% and 2.8%, respectively, by using stiffer steel cables. In the HCB-1 scheme, the outer tension cables are replaced with CFRP cables, while the inner tension cables are still made of steel cables. Due to the larger inclination angle of the inner tension cables at the girder end, its vertical component of tension is greater. The high stiffness of the steel cables effectively reduces the deflection of the main girder. Therefore, the application of hybrid cables can effectively improve the overall stiffness of the CSB compared to the CFRPCB. This characteristic is more evident at the mid-span bending moment of the main girder. The mid-span bending moment of the HCB-1 scheme is almost the same as that of the SCB, while the HCB-2 scheme is almost the same as that of the CFRPCB. Compared to the CFRPCB, the HCB-1 scheme reduces the mid-span bending moment of the main girder by 10.19%.

### 5.3. Bridge Performances Due to Temperature Load

**Figure 13:**
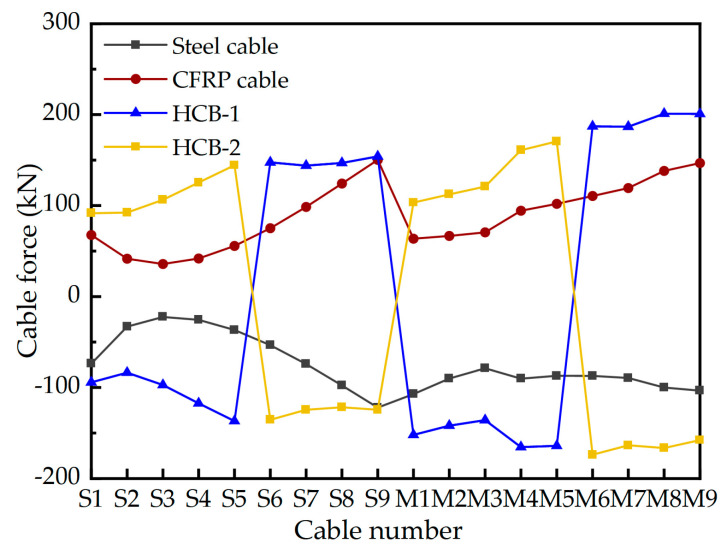
Change in cable force under temperature load. Note: The negative (positive) values of cable forces indicate that the cable force decreases (increases) under the action of temperature load.

The coefficient of thermal expansion for CFRP cables is significantly lower, only 1/17th, compared to steel cables. This means that CFRP cables are less susceptible to temperature-induced changes in force behavior compared to steel cables. Therefore, it is essential to investigate the force behavior of CFRP cables when used in CSBs under temperature load. The Specifications for Design of Highway Cable-stayed Bridge (JTG/T 3365-01-2020 [42]) outlines the design temperature combination for CSBs. This combination includes an increase in the main girder temperature by 20 °C, an increase in the bridge pylon temperature by 15 °C, and an increase in the stay cable temperature by 30 °C. Figure 13 presents the changes in cable forces for the three cable patterns under temperature load. Additionally, Table 5 provides an overview of the effects of temperature on different structural systems of the main bridge.

The results obtained from the analysis of the four CSBs exhibit significant differences, particularly in the maximum bending moments at the mid-span of the main girder. The maximum bending moments at the mid-span of the main girder for the four patterns are recorded as −9826 kN·m, 12,170 kN·m, −6388 kN·m, and 7778 kN·m, respectively. Compared to CFRPCB and SCB, both hybrid cable schemes result in a decrease in the mid-span bending moment of the main girder, with the HCB-1 scheme showing a more significant reduction, achieving a decrease of 35% and 47.5% compared to the CFRPCB and SCB respectively. Due to the thermal expansion coefficient of CFRP being only about 1/17 of steel, the deformation of CFRP cables is relatively small. It results in an increase in tension due to the mismatched deformation with the steel main girder and steel bridge pylon, causing greater upward deflection and a significant positive bending moment at the mid-span of the CFRPCB. Conversely, the greater elongation of the steel cables leads to decreased tension, causing the downward deflection of the main girders in the SCB and the generation of large negative bending moments both in the main girder span and at the base of the main pylons. Compared to the SCB and CFRPCB, the HCB exhibits the smallest mid-span bending moment and 1/4-span vertical displacement in the main girder. However, the HCB-2 scheme shows better control over the bottom bending moment of the main pylon. In conclusion, among the four cable schemes for CSBs, the HCB exhibits the least temperature effect. The HCB-1 scheme performs well in terms of the mid-span bending moment of the main girder and vertical displacement at 1/4 span, which is beneficial for enhancing the bridge’s performance under temperature loads.

## 6. Dynamic Characteristic Analysis

### 6.1. Dynamic Performances of CSBs without Considering Cables’ Local Vibrations

Determining the dynamic characteristics of the bridge is a solid basis for the structure seismic performance analysis. Using the eigenvalue analysis approach, the natural frequencies and corresponding vibration modes of the CSB based on the four cable patterns are evaluated without considering the local vibrations of the cables, and the results obtained are compared. A single truss element is employed to simulate the cables without considering the local vibrations of the cables. Table 6 lists the first ten natural frequencies and mode shapes of CSBs based on the four cable patterns without considering the cable’s local vibrations.

A brief survey of the presented results in Table 6 reveals that:

(1) Without considering the local vibrations of cables, the mode shapes of CSBs based on the four cable patterns are the same, which indicates that the variations of cable patterns have little impact on the changes in the mode shapes of the CSB.
(2) For the same vibration modes, the natural frequencies of the four patterns are similar.
(3) For the vertical bending vibrations, the order of corresponding frequencies of CSBs with various cable patterns is SCB > HCB-1 > HCB-2 > CFRPCB. This fact is mostly obvious because the vertical bending stiffness of the CSB depends on the vertical bending stiffness of the main girder and the total mass of the CSB. On the other hand, the elastic modulus of CFRP cables is much smaller than that of steel cables, which leads to a reduction in the elastic support stiffness provided by the cables to the main girder, while the mass of the CSB has a relatively limited influence on the vertical bending frequency of the bridge compared with the vertical bending stiffness of the main girder. Therefore, the exploitation of CFRP cables throughout the bridge results in the reduction of the vertical bending stiffness/mass of the girder. Compared with the CFRPCB, the implementation of hybrid cables can increase the vertical bending frequency of CSB by increasing the lateral bending stiffness of the girder.
(4) Based on the lateral bending vibrations of CSBs, we can observe the following order for the corresponding natural frequencies: CFRPCB > HCB-1 > HCB-2 > SCB. The stay cables have little influence on the lateral bending stiffness of the main girder. The lateral bending stiffness values of the main girder of the CSB based on the four cable patterns are similar, so the lateral bending frequencies of CSBs essentially depend on the mass of the CSB. Since the mass density of CFRP-based materials is about 1/5 of steel, the self-weight of the SCB is large, and the use of steel cables throughout the bridge lessens the lateral bending stiffness/mass of the main girder. Therefore, the exploitation of hybrid cables can decrease the mass of the CSB and improve the lateral bending frequency of the CSB compared with the SCB.

The vertical and lateral bending frequencies of the HCB are between those obtained for the SCB and CFRPCB. Compared with the. PCB and the SCB, the HCB improves the dynamic characteristics of the structure by enhancing the vertical bending stiffness of the main girder and lessening the self-weight of the CSB.

### 6.2. Dynamic Performances of CSBs Considering Cables Local Vibrations

To consider the influence of the cables local vibrations on the dynamic characteristics of CSBs, a multi-truss element is utilized for modeling the cables. Table 7 lists the natural frequencies and mode shapes of CSBs based on the four cable patterns in the case of considering the local vibrations of the cables.

**Figure 14:**
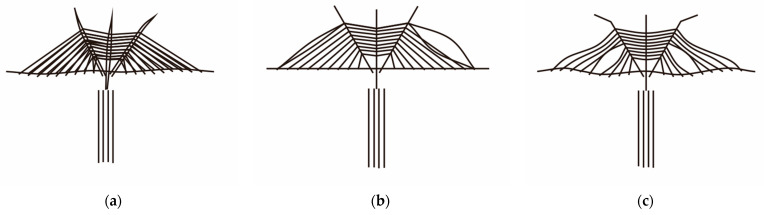
Diagrams of three categories of mode shapes after considering cables’ local vibrations: (**a**) Only the mode shapes of girder-pylon vibrations; (**b**) Only the mode shapes of the cables vibrations; (**c**) The mode shapes of the cable-bridge coupling vibrations.

The presented results in Table 8 clearly show that all vibration mode shapes of CSBs for the considered four cable patterns after considering the local vibrations of the cables can be divided into three categories (see Figure 14): (i) only the mode shapes of girder- pylon vibrations; (ii) only the mode shapes of the cables vibrations; (iii) the mode shapes of the cable-bridge coupling vibrations. Table 8 displays that the local vibration mode shapes of cables of SCB and CFRPCB first appear in the long cables, which points out that the long cables of CSBs are prone to vibrate, and the long-cable vibration of SCB appears in the low-order vibration modes, while the long-cable vibration of CFRPCB appears in the higher-order vibration modes. Because the natural frequencies of CFRP cables are much higher than that of steel cables, the appropriate arrangement of CFRP cables in the long-cable regions can delay the occurrence of long-cable vibration mode shapes and assist in the reduction of the long-cable vibrations. The short cables of the cable-stayed bridge have higher natural frequencies. Replacing the short cables with CFRP cables cannot suppress the steel cable vibrations in the long cable region, causing vibrations in the long cables at the 3rd mode shape. The cable-bridge coupling vibrations of SCB occur in lower-order modes, while cable-pylon coupling vibrations and cable-bridge coupling vibrations of CFRPCB and HCB take place in higher-order modes. It can be seen that the cable-bridge coupling vibrations are less likely to occur after the CFRP cables are arranged in the long-cable regions. The long cables of SCB have a high tendency to vibrate, which proves their fatigue failure. Therefore, the CFRP cables with good fatigue performance should be utilized in the long-cable regions.

### 6.3. Natural Vibration Characteristics of Cables

The natural frequencies of steel cables and CFRP cables in the main cable regions of CSBs are calculated using the analytical method [43]. Explicitly, the natural frequencies of an elastic cable accounting for its bending stiffness are stated:

(2) $$f_{n}=\frac{\omega_{n}}{2\pi }=\frac{n}{2L}\sqrt{\frac{T}{\rho }+\frac{n^{2}\pi^{2}EI}{\rho L^{2}}}$$

where $f_{n}$ is the n−th natural frequency, n is the vibration mode, $\omega_{n}$ is the n−th cable circle frequency, L is the calculation length of cables, T is the cable force, ρ is the density of cables, and EI denotes the bending stiffness of cables.

The study calculates and presents the first three natural frequencies of steel cables and CFRP cables in the main cable regions of the CSB in Table 8. It is observed that the first-order natural frequency of cables in the long-cable regions of the SCB falls within the range of the low-order natural frequencies of the CSB. This increases the likelihood of cable-bridge coupling vibrations and can lead to significant amplitude vibrations and fatigue damage to the cables, reducing the overall service life of the bridge. On the other hand, CFRP cables have natural frequencies that are approximately twice as high as those of steel cables and significantly exceed the low-order natural frequencies of the CSB. This means that vibrations in the CFRP cables are only likely to occur when the CSB operates at high-order vertical bending frequencies. Additionally, the natural frequencies of short steel cables are considerably higher than the low-order natural frequencies of the CSB. Based on these findings, it is concluded that the hybrid cable arrangement helps prevent cable-bridge coupling vibrations. This allocation ensures that vibrations are induced in the CFRP cables only when the CSB operates at higher vertical bending frequencies, thereby enhancing the overall safety and longevity of the bridge.

## 7. Seismic Response Analysis

According to the seismic design criteria, three seismic waves with a characteristic period of 0.55 s, the EI Centro Site wave, San Fernando wave, and Hollywood Storage P.E wave, are used as the input ground shaking, the peak accelerations of basic seismic waves in the design of horizontal and vertical directions are 0.15 *g* and 0.075 *g*, and the duration of the earthquake is 54 s. The three waves have different applicability and peak states, and their variations and locations are different. In this study, the seismic responses of CSBs are analyzed using the time-history methodology. Three different types of seismic oscillations are applied to the substructures, namely transverse + vertical, forward + vertical, and transverse + forward + vertical. The maximum values of peak internal force and peak displacement are determined at the most critical sections of the main pylon and main girder of the CSB. The results are tabulated in Table 9, providing valuable insights into the seismic behavior of CSBs under different types of seismic waves.

The results presented in Table 9 demonstrate the responses of the CFRPCB, HCB, and SCB under seismic load conditions. The maximum internal forces at the base of the main pylon and the mid-span of the main girder, as well as the maximum longitudinal and transverse displacements at the top of the main pylon and the 1/4-span of the main girder, are compared. From the analysis, it is observed that the CFRPCB and HCB exhibit similar responses, which are lower than those of the SCB. Specifically, the maximum 1/4-span vertical displacement of the CFRPCB girder is found to be 19.7% higher than that of the SCB. However, the value of the HCB is comparable to that of the SCB. These findings indicate that the HCB displays better overall performance under seismic load conditions compared to the CFRPCB and SCB. The HCB exhibits lower internal forces and displacements, suggesting improved structural integrity and resistance to seismic events. In summary, the use of hybrid cables in CSBs enhances their comprehensive performance, particularly in terms of seismic load response, as observed through reduced internal forces and displacements compared to both the CFRPCB and SCB.

The above-mentioned phenomena can be attributed to several key factors as follows:

1. In comparison with steel cables, lighter CFRP cables can decrease the self-weight of the bridge, which is beneficial to reducing the seismic response.
2. The arrangement of CFRP cables across the full span lessens the integral stiffness of the CSB, resulting in the main girder being more flexible than before. This fact leads to a larger vertical displacement response value acted upon by seismic load.
3. The natural frequencies of CFRP cables in the long-cable regions of the CSB are approximately twice as high as those of steel cables. Furthermore, the natural frequencies of CFRP cables are much larger than the lower-order natural frequencies of the CSB. This indicates that the occurrence of cable-bridge coupling vibrations is less likely when CFRP cables are used in the long-cable regions. As a result, under seismic loads, the peak seismic responses of the CFRPCB and HCB are lower than that of the SCB to varying degrees. This suggests that the utilization of CFRP cables in the CSB can effectively reduce the seismic responses experienced by the bridge.
4. The CFRP material has large damping, so CFRP cables can weaken the integral vibrations of the bridge body in the presence of seismic load.

## 8. Conclusions

The application of hybrid cables in short-span CSBs is investigated by considering the material characteristics and force forms of steel and CFRP cables. The study utilizes the finite element method to analyze the mechanical behavior of a short-span CSB under construction in China, considering four cable patterns: steel cables, CFRP cables, and two types of hybrid cables with different structural arrangements. The analysis yields the following key findings.

(1) Under the action of dead load, the use of hybrid cables can reduce the self-weight of the bridge, decrease the bending moments and deflections of the main girder, and optimize the structural performance compared with steel cable bridges. Under live load, the use of CFRP cables in the long cable region reduces the main girder axial force compared with SCB by 3.5%, which helps to improve the overall stability of the CSB; compared with CFRPCB, the use of hybrid cables reduces the main girder vertical deflection by 5.5%, which helps to improve the overall stiffness of the CSB.
(2) Under temperature load, compared with the CFRPCB and SCB, the hybrid cable pattern reduces the mid-span bending moment of the CSB by 47.5% and 35.0%, respectively. The HCB has the smallest mid-span moment and 1/4 span vertical displacement, which improves the service performance of the bridge under temperature load.
(3) The patterns of the cable system have a trivial influence on the mode shapes of the CSBs. In comparison with CFRPCB, replacing only the cables in the long cable region with CFRP cables can increase the vertical bending frequency of CSB by enhancing the vertical bending stiffness of the main girder. In comparison with SCB, replacing only the cables in the long cable region with CFRP cables can increase the lateral bending frequency of CSB by reducing the self-weight of the bridge. In the case of the same cable size, the natural frequencies of CFRP cables are about twice those of steel cables and are much larger than the lower-order natural frequencies of the entire CSB. The cable-bridge coupling vibrations are less likely to occur after the CFRP cables are arranged in the long-cable regions. Replacing the cables in the short cable region with CFRP cables cannot suppress the steel cable vibration in the long cable region or may have adverse effects on structural vibration.
(4) In the presence of seismic load, hybrid cables reduce the maximum internal forces and the maximum longitudinal and lateral displacements of the main girder and main pylon of the CSB compared with the SCB. Further, hybrid cables lessen the maximum vertical displacement of the main girder of the CSB compared with CFRPCB by 12%. Thus, the HCB has better comprehensive performances under the action of seismic load.
(5) This study uses numerical simulations to analyze the static and dynamic characteristics of HCB. By incorporating research findings on CFRP materials from various scholars, the aim is to provide reliable and realistic results. However, due to the current lack of research on the application of CFRP cables, certain factors, such as the creep issues arising from the long-term loading of steel and carbon fiber together, have not been fully considered. These issues require further research for comprehensive improvement in the future.

## Figures and Tables

**Table 1:** The boundary conditions for Tuhaihe bridge in the completed state.

Location	DX	DY	DZ	RX	RY	RZ
Fulcrum of main girder side span	×	×	×	×	×	×
Stay cables and main girders	×	×	×	×	×	×
Bridge pylons and main girders	#	#	#	Δ	Δ	Δ
Tower base and bridge pylon	#	#	#	#	#	#
Abutments and pile foundations	#	#	#	#	#	#
Pile foundation and surrounding soil	×	×	O	O	O	O

Note: (1) DX, DY, and DZ are the displacements along the X, Y, and Z directions of the bridge. RX, RY, and RZ represent the rotations around the X, Y, and Z axes, respectively. (2) Δ and # represent the degrees of freedom released and constrained by elastic or rigid connections in that direction, and O and × represent the degrees of freedom released and constrained by general supports or elastic supports in that direction.

**Table 2:** Parameters of main components.

Location	Material	Sectional Area (m^2^)	*I_Z_* (m^4^)	Density (kN/m^3^)	Modulus of Elasticity (GPa)	Coefficient of Linear Expansion
Main pylon	Q345 steel (*σ_S_* = 345 MPa)	0.342~0.700	0.532~1.096	78.5	206	1.2 × 10^−5^
Auxiliary pylon	Q345 steel (*σ_S_* = 345 MPa)	0.372~0.760	0.601~1.239	78.5	206	1.2 × 10^−5^
Pylon base	Concrete (*f_cu,k_* = 50 MPa)	55~70	114.58~145.83	25	34.5	1 × 10^−5^
Abutment	Concrete (*f_cu,k_* = 50 MPa)	277.2	6519.74	25	31.5	1 × 10^−5^
Main girder	Q345 steel (*σ_S_* = 345 MPa)	1.713~2.367	1.835~3.144	78.5	206	1.2 × 10^−5^
Pile foundation	Concrete (*f_cu,k_* = 50 MPa)	254.47	0.515	25	30.0	1 × 10^−5^
Steel cable (Per)	Steel wire (*f_pk_* = 1770 MPa)	(4.89~6.28) × 10^−3^	(1.73~3.14) × 10^−6^	78.5	205	1.2 × 10^−5^
CFRP cable (Per)	CFRP tendon (*f_pk_* = 2400 MPa)	(4.89~6.28) × 10^−3^	(1.73~3.14) × 10^−6^	16	160	7.0 × 10^−7^

**Table 3:** Meshing and sensitivity analysis.

	Mesh 1	Mesh 2	Mesh 3	Mesh 4	Mesh 5
Number of nodes	3014	2013	1543	1240	796
Number of elements	3130	2028	1561	1256	813
Minimum element size (m)	0.5	0.75	1	1.5	2
Experimental value of NW-J32	1231	1231	1231	1231	1231
Simulated value of NW-J32	1212.5	1250.7	1207.6	1194.1	1286.4
Error (%)	1.5	−1.6	1.9	3	−4.5

**Table 4:** The boundary conditions for Sutong bridge in the completed state.

Location	DX	DY	DZ	RX	RY	RZ
Tower bottom	×	×	×	×	×	×
Auxiliary pier bottom	×	×	×	×	×	×
Bridge pylon and main girder	O	×	O	O	O	O
Auxiliary pier and main girder	O	×	×	×	O	O
Stay cables and main girder	#	#	#	#	#	#

Note: (1) DX, DY, and DZ are the displacements along the X, Y, and Z directions of the bridge. RX, RY, and RZ represent the rotations around the X, Y, and Z axes, respectively. (2) # represent the degrees of freedom released and constrained by rigid connections in that direction, and O and × represent the degrees of freedom released and constrained by general supports or elastic supports in that direction.

**Table 5:** Temperature effects of different structural systems of the bridge.

Sectional Position (Force or Displacement)	SCB	CFRPCB	HCB-1	HCB-2
Mid span bending moment of main girder (kN·m)	−9826	12,170	−6388	7778
Vertical displacement of main girder 1/4 span (mm)	−8	13	3	4
Horizontal displacement of girder end (mm)	−24	−24	−24	−24
Bending moment at the base of main pylon (kN·m)	−3057.5	982.1	−1831.7	−431.3
Displacement at the top of main pylon (mm)	−11	−8	−9	−10

**Table 6:** Natural frequencies and mode shapes of each bridge type are not considered without considering the local vibrations of the cables.

Order of Mode	Frequencies (Hz)				Mode Shapes
	SCB	CFRPCB	HCB-1	HCB-2	
1	0.8716	0.8458	0.8621	0.8546	1st anti. V (girder)
2	1.3050	1.2344	1.2776	1.2624	1st sym. V (girder)
3	1.6680	1.6642	1.6654	1.6674	LF (girder), LB (main pylon and auxiliary pylon)
4	1.7297	1.7670	1.7574	1.7389	1st L (main pylon and girder)
5	1.9304	1.9761	1.9656	1.9404	anti. L (main pylon)
6	2.4926	2.4271	2.4598	2.4554	LB (main pylon)
7	2.6459	2.6912	2.6845	2.7033	2nd L (main pylon and girder)
8	2.7358	2.7372	2.7301	2.7768	2nd V (girder), LB (main pylon)
9	2.7798	2.7427	2.7399	3.0598	LB (main pylon and auxiliary pylon)
10	3.1055	3.0487	3.0948	3.5430	3rd V (girder), LB (main pylon)

Note: L—lateral bending; V—vertical bending; LF—longitudinal floating; LB—longitudinal bending; sym.—symmetric; anti.—antisymmetric.

**Table 7:** Natural frequencies and mode shapes of each bridge type considering cables’ local vibrations.

	Order	*f* (Hz)	Mode Shapes
SCB	1–2	0.8721–1.3054	girder-pylon vibrations
	3	1.6639	cable-bridge coupling vibrations
	4–21	1.6712–2.4840	long-cable vibrations
	38	2.4947	cable-bridge coupling vibrations
CFRPCB	1–12	0.8459–3.6090	girder-pylon vibrations
	13	3.6710	cable-pylon coupling vibrations
	14–21	3.7937–3.7974	long-cable vibrations
	32	4.3123	cable-bridge coupling vibrations
HCB-1	1–9	0.8623–2.7401	girder-pylon vibrations
	10–17	2.9021–2.9127	cable vibrations
	18	3.0951	steel cable-bridge coupling vibrations
	19	3.4951	steel cable-pylon coupling vibrations
HCB-2	1–2	0.8549–1.2628	girder-pylon vibrations
	3	1.6634	long-cable vibrations
	4–28	1.6744–2.1597	cable-bridge coupling vibrations
	29–32	2.1695–2.4723	steel cable-pylon coupling vibrations

**Table 8:** Cable fundamental frequencies of the CSB (Units: Hz).

NO.	Steel Cable Fundamental Frequencies (Hz)			CFRP Cable Fundamental Frequencies (Hz)		
	1st Order	2nd Order	3rd Order	1st Order	2nd Order	3rd Order
S1	7.6152	15.441	23.687	16.840	34.049	51.982
S2	5.7978	11.671	17.692	12.828	25.785	39.000
S3	4.4590	8.9489	13.500	9.8667	19.787	29.813
S4	3.5674	7.1497	10.762	7.8938	15.814	23.785
S5	2.9462	5.9006	8.7814	6.5188	13.052	19.613
S6	2.4981	5.0012	7.5142	5.5270	11.063	16.616
S7	2.1694	4.3420	6.5211	4.7993	9.6041	14.420
S8	1.9203	3.8429	5.7698	4.2480	8.4997	12.759
S9	1.7229	3.4474	5.1750	3.8109	7.6245	11.443

**Table 9:** Maximum internal forces and displacements of the most unfavorable sections of the main pylon and girder.

Sectional Positions (Forces or Displacements)		SCB	CFRPCB (*R_CS_*)	HCB-1 (*R_HS_*; *R_HC_*)	HCB-2 (*R_HS_*; *R_HC_*)
Main pylon	Axial forces at pylon base (kN)	696	660 (−5.2%)	669 (−3.9%; 1.4%)	673 (−3.3%; 2.0%)
	Transverse shearing forces at pylon base (kN)	2328	2295 (−1.4%)	2306 (−1.0%; 0.5%)	2314 (−0.6%; 0.8%)
	Longitudinal shearing forces at pylon base (kN)	2860	2548 (−10.9%)	2731 (−4.5%; 7.2%)	2559 (−4.5%; 7.2%)
	Transverse bending moments M_x_ at pylon base (kN·m)	2415	2304 (−4.6%)	2323 (−3.8%; 0.8%)	2299 (−10.5%; 0.43%)
	Longitudinal bending moments M_y_ at pylon base (kN·m)	16,364	16,103 (−1.6%)	16,249 (−0.7%; 0.9%)	16,257 (−0.7%; 0.95%)
	Transverse bridge displacements on pylon top (mm)	68.164	67.023 (−1.7%)	67.274 (−1.3%; 0.4%)	67.383 (−1.1%; 0.5%)
	Bridge longitudinal displacements on pylon top (mm)	44.667	41.54 (−7.0%)	41.898 (−6.2%; 0.9%)	42.12 (−5.7%; 1.4%)
Main girder	Mid-span axial forces (kN)	18,179	17,486 (−3.8%)	17,763 (−2.3%; 1.6%)	17,867 (−1.7%; 2.1%)
	Mid-span transverse shearing forces (kN)	7594	7316 (−3.7%)	7354 (−3.2%; 0.5%)	7355 (−3%; 0.5%)
	Mid-span vertical shearing forces (kN)	1927	1905 (−1.1%)	1908 (−1.0%; 0.2%)	1923 (−0.2%; 0.9%)
	Mid-span transverse bending moments (kN·m)	285,691	281,569 (−1.4%)	282,575 (−1.1%; 0.4%)	284,385 (−0.5%; 1.0%)
	Mid-span vertical bending moments (kN·m)	42,701	41,824 (−2.1%)	42,075 (−1.5%; 0.6%)	42,645 (−0.1%; 2.0%)
	1/4-span transverse bridge displacements (mm)	12.668	12.516 (−1.2%)	12.581 (−0.7%; 0.5%)	12.636 (−0.3%; 1.0%)
	1/4-span longitudinal bridge displacements (mm)	35.733	35.103 (−1.8%)	35.258 (−1.3%; 0.4%)	35.582 (−0.4%; 1.4%)
	1/4-span vertical displacements (mm)	34.076	40.797 (19.7%)	35.884 (5.3%; −12%)	37.551 (10.2%; −8.0%)

Note: *R_CS_*, *R_HS_*, and *R_HC_* represent the growth rate of the forces or displacements of the CFRPCB relative to the SCB, HCB relative to SCB, and HCB relative to CFRPCB, respectively.

## Data Availability

The data presented in this study are available on request from the authors.
